# Association of dietary total antioxidant capacity, alternative healthy eating index, and dietary inflammatory index with semen quality in men seeking infertility treatment

**DOI:** 10.3389/fnut.2023.1284379

**Published:** 2023-10-11

**Authors:** Mahtab Dabagh, Nargesbano Jahangiri, Ali Taheri Madah, Sahar Rostami, Fardin Amidi, Mahshad Khodarahmian, Ali Tavoosian, Maryam Shabani Nashtaei, Akram Vatannejad

**Affiliations:** ^1^Department of Anatomy, School of Medicine, Tehran University of Medical Sciences, Tehran, Iran; ^2^Department of Gynecology and Obstetrics, Yas Hospital Complex, Tehran University of Medical Sciences, Tehran, Iran; ^3^Department of Gynecology and Obstetrics, Arash Women’s Hospital, Tehran University of Medical Sciences, Tehran, Iran; ^4^Department of Urology, School of Medicine, Tehran University of Medical Sciences, Tehran, Iran; ^5^Department of Infertility, Shariati Hospital, Tehran University of Medical Sciences, Tehran, Iran; ^6^Department of Comparative Biosciences, Faculty of Veterinary Medicine, University of Tehran, Tehran, Iran

**Keywords:** infertility, semen analysis, nutrients, dietary total antioxidant capacity (dTAC), alternative healthy eating index (AHEI), dietary inflammatory index (DII)

## Abstract

**Background:**

Since the association between dietary quality scores and semen quality remains unclear, we carried out a hospital-based cross-sectional study to investigate the association of Dietary Total Antioxidant Capacity (dTAC), Dietary Inflammatory Index (DII), and Alternative Healthy Eating Index (AHEI) scores with semen quality in men seeking infertility treatment.

**Methods:**

This study enrolled 210 men with unexplained or idiopathic infertility. Semen samples were collected and analyzed according to the WHO 2010 criteria. Dietary data was collected using a 168-item semi-quantitative food frequency questionnaire (FFQ) developed for Tehran Lipid and Glucose Study. Multivariable logistic regression models were used to estimate the relationship between dTAC, AHEI, and DII scores with abnormal semen in crude and adjusted models.

**Results:**

There were no significant differences across quartile categories of the dTAC, AHEI, and DII scores regarding semen parameters. There was a trend toward a significant direct association between DII and abnormal semen risk (*p* = 0.01). Infertile men in the highest quartile of DII had a 2.84 times higher risk of abnormal semen in the crude model (OR: 3.84; 95% CI: 1.64–8.95); such that remained after adjusting for several potential confounders. There was no significant association between dTAC or AHEI and the risk of abnormal semen in infertile men, either before or after adjusting for potential confounders. Total energy (*p* = 0.05), fat (*p* = 0.02), saturated fat (*p* = 0.02), mono-saturated fat (*p* = 0.009), Thiamine (Vitamin B1) (*p* = 0.02), Niacin (Vitamin B3) (*p* = 0.03), Calcium (*p* = 0.01), and Selenium (*p* = 0.01) were inversely associated with semen normality.

**Discussion:**

The study suggests that certain dietary factors may affect semen quality, and the mechanisms underlying the observed associations are likely multifactorial, involving complex interactions between diet, oxidative stress, inflammation, and hormone levels. Further research is required to confirm the results, fully elucidate the mechanisms underlying the associations, and identify specific dietary interventions that may improve male fertility outcomes.

## Introduction

Infertility, estimated to impact 8%–12% of couples who are in their reproductive years, is a major global health issue (1). Based on a survey conducted by the Global Burden of Disease, it was found that from 1990 to 2017, there was a yearly increase of 0.370% in the age-standardized prevalence of infertility among women, and a 0.291% increase among men (2). While several factors can contribute to male infertility, including genetic abnormalities and medical conditions, modifiable environmental and lifestyle factors, including diet have also been implicated to play a key role in the etiology of infertility (3). Among these, diet has emerged as a potential modifiable risk factor (4).

Recently, there has been an increasing focus on the impact of dietary patterns and nutrients on the quality of semen and fertility status. These factors serve as indicators of a man’s overall health (5). According to numerous studies which have investigated the relationship between diet and infertility, a diet rich in fruits, vegetables, whole grains, and lean proteins, as well as micronutrients, such as antioxidants and omega-3 fatty acids, may improve semen quality (6–8), while a diet rich in processed foods, meats, sugar, and saturated fat can have adverse effects on the quality of semen (9–14).

Excessive amounts of reactive oxygen species (ROS), frequently present in the semen of men experiencing infertility, can lead to harm to the sperm membrane through the initiation of lipid peroxidation, an abnormal increase in membrane fluidity, chromatin oxidation, and the production of advanced glycation end products (AGEs). These effects ultimately lead to a reduction in fertility and can negatively affect the parameters of semen quality (15, 16). However, endogenous antioxidants together with exogenous antioxidants obtained from the diet can neutralize some ROS-induced oxidative damage (17). Limited information is available on how the levels of commonly consumed antioxidants from dietary sources affect semen quality (18, 19). There have been only a few observational studies that have examined the connection between the intake of dietary antioxidants and the quality of semen, all of which were performed on healthy men, and all showed a positive association between dietary antioxidants and semen quality (20, 21). On the other hand, assessing the antioxidant capacity of a diet and understanding the combined effects of various dietary antioxidants is crucial, as relying solely on the evaluation of individual antioxidant compounds fails to provide a comprehensive picture (22, 23). Thus, the dietary total antioxidant capacity (dTAC) can be used to assess the antioxidant capacity of the diet, which has a close relationship with a healthy diet (24).

The Healthy Eating Index (HEI), the alternate Mediterranean Diet score (aMED), the Alternative Healthy Eating Index (AHEI), and the Dietary Approaches to Stop Hypertension (DASH) are widely used nutritional indices commonly used to represent and reflect major nutritional patterns. Researchers have studied the relationship between these dietary pattern indices and semen parameters (25). The best results have been obtained by following the AHEI-2010, which had a significant positive impact on sperm quality parameters.

Diet is a crucial factor in regulating inflammation and has a key role in the onset and progression of male infertility (26). The dietary inflammatory index (DII) is a measure that takes into account six markers of inflammation (TNF-α, C-reactive protein (CRP), interleukin (IL)-1β, IL-4, IL-6, and IL-10) and their relationship to various dietary factors like flavonoids, vitamins, macronutrients, minerals, and specific foods (27–29). Research has shown that the DII is associated with chronic illnesses such as metabolic syndrome (30), cardiovascular disease (31), and cancer (32). DII has been discovered to have an impact on men’s reproductive health, as those who follow a diet that promotes inflammation seem to be at a greater risk of experiencing low testosterone levels (33). However, controversial findings have been reported on the association between DII and male reproductive parameters (26, 34).

Since the association between these dietary indicators and semen quality remains unclear, we designed a cross-sectional study to examine the association of dTAC, AHEI, and DII with semen quality in men seeking infertility treatment.

## Materials and methods

### Patient enrollment and study design

In this cross-sectional study, we studied the relationship between dietary quality scores and sperm quality in 210 men with unexplained or idiopathic infertility seeking assisted reproductive techniques (ARTs) at the referral infertility clinics of Arash and Dr. Shariati hospitals affiliated with Tehran University of Medical Sciences (TUMS), Tehran, Iran, between 2020 and 2022. Male patients aged 20–40 with a normal reproductive and medical history, normal findings on physical examination, and genetic and hormonal evaluation were included. The participants had no long-term dietary restrictions as well. However, their semen analysis revealed normal or abnormal parameters with failure to achieve fatherhood despite a minimum of 1 year of regular unprotected sexual intercourse. An experienced andrologist performed all clinical and physical examinations. Infertile men with azoospermia and couples with and couples who experienced infertility due to female factors were excluded from the study.

### Semen sample collection and analysis

Men unable to conceive a child naturally after one year of regular unprotected sexual intercourse and seek help from infertility clinics are examined by a specialist called an andrologist. After obtaining written informed consent a part of the semen was used for the current study. Semen parameters, including volume, sperm concentration, motility, and morphology, were analyzed. The collection, examination, and processing of semen were performed based on the WHO laboratory manual for the examination and processing of human semen, 5th edition (35).

### Demographic and dietary data collection

Patients’ demographic data [age, BMI, waist-to-hip ratio (WHR)], education, smoking history, and supplement use were recorded. In addition, the 168-item semi-quantitative food frequency questionnaire (FFQ) developed for Tehran Lipid and Glucose Study and validated by Esfahani et al. (36, 37) was administered face-to-face by trained interviewers for dietary assessments for the past 12 months.

The study participants were asked to indicate how frequently they consumed each food item, whether it was on a daily, weekly, or monthly basis. The patients shared information about their food consumption over the past year. In cases where participants could not select a consumption frequency from the provided options in the questionnaire, they provided their own portion sizes. These portion sizes were then adjusted to match the portion sizes mentioned in the questionnaire. The daily portion size of the reported consumed foods was calculated and converted to grams. To evaluate food intake, a software called Nutritionist IV (First Databank Division, the Hearst Corporation, San Bruno, CA, United States) was used, which had been modified to accommodate Iranian foods.

### Semen collection, preparation, and analysis

Fresh semen samples were obtained through masturbation in sterile polypropylene containers after three to five days of abstention from sexual activity. The samples were examined in the lab for 30 to 40 min. The samples were kept at 37°C for 30 min within an hour to achieve liquefaction, and semen parameters (volume, concentration, total sperm count, percentage of motility, and percentage of normal morphology) were evaluated according to the WHO laboratory manual for the examination and processing of human semen, 5th edition (35). The WHO reference values for normal semen include semen volume > 1.5 mL, semen concentration > 15 × 10^6^ cells/mL, total sperm >39 × 10^6^, motility >40%, and normal semen morphology >4% (35). Semen volume > 1.5 mL, semen concentration > 15 × 10^6^ cells/mL, total sperm >39 × 10^6^, motility >40%, and normal semen morphology >4%. The WHO standards were utilized to calculate the proportion of sperm with normal morphology. Sperm morphology was visualized by using a Diff-Quick staining kit (RS Medical, Ravan Sazeh Co., Iran) according to the manufacturer’s instructions. A 10-mL sample of sperm was placed in a Makler chamber (Sefi-Medical Instruments) to measure sperm concentration and motility (38, 39). Semen was then seen using a phase-contrast microscope (Olympus CX21) at 200x. Using the volume concentration equation, the total sperm count was calculated. Experienced technicians conducted all analyses, and external quality control was consistently implemented throughout the study.

### Measurement of anthropometric indices

The participants’ weight was assessed by using digital scales while they were minimally clothed and not wearing shoes. The data was recorded with an accuracy of 1 kg. Height was measured while standing without shoes, ensuring that the shoulders were in a natural position. To determine the Body Mass Index (BMI), the weight in kilograms was divided by the square of the height (m^2^).

### Assessment of diet scores

The FFQ-derived dietary data was used to calculate dTAC, DII, and AHEI scores for all of the patients. The DII score was used to measure the inflammatory effect of a person’s diet. It has been proven to be accurate in determining the impact on various inflammatory markers. This score is used to calculate the overall inflammatory potential of the diet (40). The dTAC was calculated by multiplying the daily consumption of each chosen food item by its specific antioxidant value per serving, and then adding up the resulting values. Antioxidants from supplements were not included (41). The AHEI assigns scores to different components of the diet based on their potential health benefits, with higher scores indicating a healthier overall diet. The components that are scored include vegetables, fruits, whole grains, nuts and legumes, polyunsaturated fatty acids (PUFAs), omega-3 fatty acids, moderate alcohol consumption, red and processed meat consumption, sugary drinks and fruit juice consumption, sodium intake (42). Studies have shown that diets that score high on AHEI and dTAC, and low on DII are associated with protective effects against many chronic diseases.

### Statistical analysis

We divided all participants into groups based on quartiles of their dTAC, AHEI, and DII scores. Differences in variables across quartiles of dTAC, AHEI, and DII scores, according to normality distribution, were tested with the use of one-way variance or the Kruskal–Wallis test. The Chi-square test was applied assessment of categorical variables. BMI was categorized into normal weight (<25 kg/m^2^) or overweight and obese (≥25 kg/m^2^). Patients were categorized based on smoking history into non-smokers or smokers. They were also classified into two categories (bachelor’s degree or higher; and high school diploma or less) according to educational level, and based on supplement use (yes vs. no).

Multivariate logistic regression models were used to estimate odds ratios (ORs) and corresponding 95% confidence intervals (CIs) to assess associations of dTAC, AHEI, and DII scores with abnormal semen risk in crude and adjusted models. In Model I, we only adjusted for total calorie intake (kcal/day). In the second regression model (Model II), we further adjusted for age (years) and BMI (kg/m2). In Model III, we further adjusted for smoking history, supplement use, and educational status.

Nutrient items were reported in the total study population. Also, infertile men were stratified by normal (*n* = 123) and abnormal (*n* = 87) semen analysis and nutrient items compared using the student *t*-test. Data analysis was carried out using SPSS v22.0 software (Inc., Chicago, IL). A *p*-value of less than 0.05 was considered significant.

### Ethical approval

The study was approved by the Deputy of the Research and Ethics Committee of TUMS (approval date: 2020, 02, 05; code: IR.TUMS.MEDICINE.REC.1398.858) and carried out in accordance with the Declaration of Helsinki, the International Council for Harmonization Guidelines for Good Clinical Practice, and relevant regulatory requirements.

## Results

Table 1 presents a comparison of sperm parameters between two groups: the “Normal Semen” group (consisting of 123 male patients) and the “Abnormal Semen” group (consisting of 87 men). The main characteristics of the individuals involved in the study, is categorized based on quartiles of the dTAC, AHEI, and DII scores (Table 2). The median dTAC score was 120.56 ± 162.95 in the total population. Higher dTAC scores were associated with higher total energy intake (*p* = 0.01). There were no other significant differences across categories of the dTAC score. The median AHEI score was 49.48 ± 9.8, which was significantly higher in men with abnormal semen (48.34 ± 10.27 vs. 51.09 ± 8.89, *p* = 0.04). Although not statistically significant, the WHR was inversely associated with the AHEI score (*p* = 0.07). While those with higher energy intake had a higher AHEI score (*p* = 0.000). No significant differences were observed among the AHEI score categories. The median DII score was −3.05 ± 0.95, which was significantly higher in men with abnormal semen (−3.17 ± 0.91 vs. -2.89 ± 0.99; *p* = 0.03). There were no differences between categories of DII in BMI, smoking history, and supplement use. The reported age, WHR, and education tended to decrease significantly with increasing categories of DII (*p* = 0.01, 0.05, and 0.04 respectively). While higher DII scores were associated with higher total energy intake (*p* < 0.001).

**Table 1:** Comparison of sperm parameters between men with normal and abnormal semen.

Sperm parameters	Total population		*p*-value
	Normal semen (*n* = 123)	Abnormal semen (*n* = 87)	
Concentration (×10^6^/ml)	33.4228 ± 13.11	19.56 ± 17.91	****<0.001
Total motility (%)	48.79 ± 14.83	32.58 ± 20.21	****<0.001
Normal morphology (%)	5.47 ± 1.6	2.04 ± 1.25	****<0.001
Volume (ml)	3.05 ± 1.65	2.68 ± 1.11	0.07

Data are presented as mean ± SD. A *p*-value of < 0.05 was considered significant. ***p < 0.001.

**Table 2:** The main characteristics of the study participants according to quartiles of the dTAC, AHEI, and DII score.

Index	dTAC					AHEI					DII				
Characteristics	Q1	Q2	Q3	Q4	*p*-value	Q1	Q2	Q3	Q4	*p*-value	Q1	Q2	Q3	Q4	*p*-value
**Score**	≤19.15	20.7–28.38	93.25–152.02	≥187.84		≤29	44–49	51–53	≥58		≤ − 3.88	−3.66 – −3.08	−2.89 – –2.81	≥ − 2.23	
**Age (years)**	35 (32–40)	36 (33–39)	37 (32.25–40)	36 (33–39.75)	0.9	37 (33–41)	36 (32–39.25)	36 (32–39)	35 (33–40)	0.27	37 (34–41)	37 (32–40)	36 (32–39)	35 (31–38)	*0.01
**Total energy**															***<0.001
**Intake (kcal/d)**	2444.25 (2088.88–2920.08)	2558.18 (2148.81–3384.06)	2869.17 (2241.57–3489.32)	2811.2 (2332.36–3598.15)	*0.01	1887.88 (1516.62–2287.43)	2599.24 (2103.78–3055.57)	2714.11 (2187.99–3271.92)	3533.98 (2891.28–4480.35)	****0.000	2516.59 (1857.96–3056.59)	2467.6 (1858.50–3002.57)	2714.1 (2187.99–3271.92)	3066.79 (2558.18–3871.62)	
**WHR**	0.89 (0.9–0.93)	0.89 (0.9–0.93)	0.9 (0.89–0.92)	0.9 (0.89–0.94)	0.96	0.92 (0.89–0.95)	0.90 (0.89–0.93)	0.90 (0.89–0.92)	0.90 (0.88–0.92)	0.07	0.91 (0.89–0.94)	0.90 (0.88–0.95)	0.90 (0.89–0.93)	0.89 (0.88–0.92)	0.05
**BMI,** kg/m2	26.81 (24.83–28.4)	27.68 (25.16–28.72)	28.07 (25.21–29.92)	27.15 (24.84–29.53)	0.53	27.46 (26.15–28.88)	27.47 (24.97–30.14)	27.23 (24.81–29.39)	27.44 (24.80–29.75)	0.91	27.78 (26.13–29.75)	27.39 (24.72–29.74)	27.47 (25.68–29.05)	26.51 (24.1–28.73)	0.07
**BMI category**															0.13
Normal weight (<25 kg/m2)	14 (26.4)	13 (24.5)	12 (22.6)	14 (26.4)	0.96	12 (22.6)	12 (22.6)	15 (28.3)	14 (26.4)	0.89	9 (17)	15 (28.3)	11 (20.8)	18 (34)	
Overweight and obese (≥25 kg/m2)	38 (24.4)	40 (25.6)	40 (25.6)	38 (24.4)		41 (26.3)	38 (24.4)	37 (23.7)	40 (25.6)		44 (28.2)	37 (23.7)	42 (26.9)	33 (21.2)	
**Smoking history**															0.23
Nonsmoker	41 (25.8)	44 (27.7)	39 (24.5)	35 (22)	0.21	39 (24.5)	41 (25.8)	39 (24.5)	40 (25.2)	0.84	40 (25.2)	45 (28.3)	36 (22.6)	38 (23.9)	
Smoker	11 (21.6)	9 (17.6)	13 (25.5)	18 (35.3)		14 (27.5)	10 (19.6)	13 (25.5)	14 (27.5)		13 (25.5)	8 (15.7)	17 (33.3)	13 (25.5)	
**Supplement use**															0.73
Yes	25 (21.4)	27 (23.1)	33 (28.2)	32 (27.4)	0.32	30 (25.6)	25 (21.4)	32 (27.4)	30 (25.6)	0.64	28 (23.9)	31 (26.5)	32 (27.4)	26 (22.2)	
No	27 (29)	26 (28)	19 (20.4)	21 (22.6)		23 (24.7)	26 (28)	20 (21.5)	24 (25.8)		25 (26.9)	22 (23.7)	21 (22.6)	25 (26.9)	
**Education status**															*0.04
Bachelor degree or higher	15 (20.5)	15 (20.5)	18 (24.7)	25 (34.2)	0.14	23 (31.5)	18 (24.7)	17 (23.3)	15 (20.5)	0.39	22 (30.1)	24 (32.9)	16 (21.9)	11 (15.1)	
High school diploma or less	37 (27)	38 (27.7)	34 (24.8)	28 (20.4)		30 (21.9)	33 (24.1)	35 (25.5)	39 (28.5)		31 (22.6)	29 (21.2)	37 (27)	40 (29.2)	

Values are presented as median (interquartile range), or *n* (%). Differences in variables across all four quartiles in the indexes were tested with the use of Kruskal–Wallis test for continuous variables and chi-square test for categorical variables. dTAC = dietary total antioxidant capacity; AHEI, alternative healthy eating index; DII, dietary inflammation index. *p < 0.05, **p < 0.01, ***p < 0.001, ****p < 0.0001.

We also compared total energy and the indices between men with normal and abnormal semen (Table 3). The *p*-value of 0.05 suggests a marginally significant difference in energy levels between the two groups. Statistically significant differences in DII (*p* = 0.03) and AHEI (*p* = 0.04) were observed between the two groups. However, we found no significant differences between men with normal and abnormal semen regarding dTAC (*p* = 0.7).

**Table 3:** Comparison of total energy, DII, AHEI, and dTAC between men with normal and abnormal semen.

Sperm parameters	Total population	Total population		*p*-value
		Normal semen (*n* = 123)	Abnormal semen (*n* = 87)	
**Energy** (Kcal)	2841.03 ± 1055.78	2721.05 ± 1085.49	3010.66 ± 993.81	0.05
**DII**	−3.05 ± 0.95	−3.17 ± 0.91	−2.89 ± 0.99	*0.03
**AHEI**	49.48 ± 9.8	48.34 ± 10.27	51.09 ± 8.89	*0.04
**dTAC**	120.56 ± 162.95	123.15 ± 154.6	116.91 ± 174.93	0.7

Values are presented as mean ± SD. A *p*-value of < 0.05 was considered significant. *p < 0.05.

As shown in Table 4, there were no significant differences across categories of the dTAC, AHEI, and DII scores regarding semen parameters. While there was a negative correlation between the DII and semen volume (*p* = 0.09), the difference was not statistically significant.

**Table 4:** Participants’ semen parameters across categories of the dTAC, AHEI, and DII scores.

Index	dTAC					AHEI					DII				
Semen parameters	Q1	Q2	Q3	Q4	*p*-value	Q1	Q2	Q3	Q4	*p*-value	Q1	Q2	Q3	Q4	*p*-value
Volume (ml)	2.71 ± 1.1	2.99 ± 1.85	2.9 ± 1.2	2.99 ± 1.59	0.74	2.66 ± 1.13	2.89 ± 1.46	2.86 ± 1.42	3.18 ± 1.76	0.34	3.15 ± 2.1	3.13 ± 1.26	2.76 ± 1.03	2.54 ± 1.15	*0.09
Concentration (×10^6^/ml)	23.94 ± 14.77	30.41 ± 16.45	26.36 ± 18.67	29.90 ± 16.33	0.15	31.11 ± 15.55	28.03 ± 18.35	26.86 ± 15.6	24.75 ± 17.03	0.25	30.37 ± 13.26	28.09 ± 19.4	27.45 ± 15.5	24.68 ± 18.04	0.38
Total motility (%)	39.46 ± 17.5	43.75 ± 19.85	41.28 ± 20.74	43.75 ± 17.88	0.59	42.09 ± 16.8	43.03 ± 20.02	40.98 ± 18.3	42.22 ± 21.01	0.95	46.03 ± 16.04	39.45 ± 16.45	41.81 ± 21.26	40.98 ± 21.49	0.32
Normal morphology (%)	3.80 ± 2.51	4.26 ± 2.32	3.96 ± 2.23	4.16 ± 1.88	0.72	4.56 ± 2.56	4.11 ± 2.09	3.82 ± 1.94	3.7 ± 2.26	0.19	4.6 ± 2.02	3.94 ± 2.31	4.07 ± 2.17	3.56 ± 2.37	0.12

*p*-values were determined by the ANOVA test. Data are presented as mean ± SD. *p < 0.05.

Associations between diet indices scores and abnormal semen risk are shown in Table 5. There was no statistically significant association between dTAC with the abnormal semen in any quartile or model. For AHEI, there was a borderline statistically significant association in Q1 in Model I (OR = 2.08, 95% CI 0.92–4.69, *p* = 0.09), but the association was not remained after adjusting for age, BMI, and other confounders in Models II and III. There was a trend towards significant direct association between DII and abnormal semen risk (*p* = 0.01). We found that infertile men in the highest quartile of DII had a 2.84 times higher risk of abnormal semen in the crude model (OR: 3.84; 95% CI: 1.64–8.95). This association endured significant after further adjusting for other potential confounders in model I, II, and III.

**Table 5:** Risk for abnormal semen according to quartiles of the dTAC, AHEI, DII scores.

	Crude	Model I	Model II	Model III
dTAC				
Q1	1.00 (Ref)	1.00 (Ref)	1.00 (Ref)	1.00 (Ref)
Q2	0.7 (0.32–1.54)	0.64 (0.2–1.42)	0.66 (0.3–1.47)	0.64 (0.29–1.44)
Q3	0.92 (0.42–2)	0.78 (0.35–1.74)	0.83 (0.37–1.87)	0.84 (0.37–1.92)
Q4	0.7 (0.32–1.54)	0.58 (0.26–1.31)	0.63 (0.28–1.42)	0.64 (0.27–1.48)
*p*-value	0.5	0.2	0.3	0.4
AHEI				
Q1	1.00 (Ref)	1.00 (Ref)	1.00 (Ref)	1.00 (Ref)
Q2	2.08 (0.92–4.69)	1.79 (0.76–4.2)	1.83 (0.77–4.35)	0.74 (0.26–2.14)
Q3	2.34 (1.04–5.26)	1.9 (0.78–4.61)	1.79 (0.73–4.39)	1.29 (0.53–3.14)
Q4	2.02 (0.9–4.52)	1.39 (0.5–3.91)	1.4 (0.49–3.96)	1.33 (0.58–3.06)
*p*-value	0.09	0.5	0.5	0.6
DII				
Q1	1.00 (Ref)	1.00 (Ref)	1.00 (Ref)	1.00 (Ref)
Q2	3.54 (1.53–8.21)	3.54 (1.52–8.24)	3.52 (1.49–8.28)	3.44 (1.45–8.14)
Q3	2.24 (0.96–5.22)	2.16 (0.92–5.06)	2 (0.84–4.73)	2.14 (0.89–5.12)
Q4	3.84 (1.64–8.95)	3.3 (1.38–7.9)	2.86 (1.17–6.99)	2.93 (1.18–7.24)
*p*-value	*0.01	*0.03	0.08	0.06

Model I is adjusted for total calorie intake (kcal/day). Model II is adjusted for total calorie intake (kcal/day), age (years) and BMI (kg/m^2^). Model III is adjusted for total calorie intake (kcal/day), age (years) and BMI (kg/m^2^), smoking history, supplement use and educational status. *p < 0.05.

Table 6 shows significant differences in the level of total energy (*p* = 0.05), fat (*p* = 0.02), saturated fat (*p* = 0.02), mono-saturated fat (*p* = 0.009), Thiamine (Vitamin B1) (*p* = 0.02), Niacin (Vitamin B3) (*p* = 0.03), Calcium (*p* = 0.01), and Selenium (*p* = 0.01) between infertile men with and without normal semen.

**Table 6:** Comparison of nutrient items between men with normal and abnormal semen.

Nutrient items	Total population	Total population		*p*-value
		Normal semen (*n* = 123)	Abnormal semen (*n* = 87)	
Energy (kcal/d)	2841.03 ± 1055.78	2721.05 ± 1085.49	3010.66 ± 993.81	0.05
Protein	100.08 ± 39.81	96.48 ± 40.86	105.16 ± 37.92	0.12
Carbohydrate	411.85 ± 156.25	398.21 ± 157	431.14 ± 154.03	0.13
Fat	88.14 ± 42.98	82.47 ± 42.78	96.15 ± 42.22	*0.02
Dietary fiber	20.95 ± 9.74	7.38 ± 4.4	8.59 ± 8.1	0.17
Cholesterol	88.14 ± 42.98	344.19 ± 202.84	388.06 ± 210.35	0.13
Saturated fat	27.78 ± 16.10	25.71 ± 15.17	30.69 ± 16.99	*0.02
Mono_fat	24.94 ± 13.96	22.85 ± 13.66	27.9 ± 13.93	**0.009
Poly_fat	17.50 ± 9.41	16.68 ± 9.83	18.66 ± 8.71	0.13
Vitamin A	1589.27 ± 1569.39	1585.4 ± 1603.03	1594.74 ± 1529.76	0.96
Beta carotene	955.55 ± 1415.89	948.82 ± 1424.64	965.06 ± 1411.62	0.93
Vitamin E	12.09 ± 9.58	4.34 ± 2.17	4.78 ± 2	0.96
Thiamine (Vitamin B1)	2.21 ± 0.79	2.11 ± 0.84	2.36 ± 0.7	*0.02
Riboflavin (Vitamin B2)	1.65 ± 0.77	1.56 ± 0.74	1.76 ± 0.8	0.06
Niacin (Vitamin B3)	25.8 ± 9.38	24.68 ± 9.5	27.4 ± 9.03	*0.03
Pantothenic acid (Vitamin B5)	6.2 ± 2.76	5.92 ± 2.72	6.59 ± 2.78	0.08
Vitamin B6	1.71 ± 0.86	1.63 ± 0.86	1.82 ± 0.85	0.11
Vitamin B12	4.52 ± 3.96	4.52 ± 4.58	4.51 ± 2.9	0.98
Vitamin C	184.68 ± 112.76	187.73 ± 124.26	180.36 ± 94.68	0.64
Vitamin D	1.74 ± 1.56	1.63 ± 1.49	1.88 ± 1.65	0.25
Biotin	29.96 ± 12.28	28.9 ± 12.6	31.4 ± 11.7	0.13
Folate	352.41 ± 168.03	344.22 ± 167.1	364 ± 169.62	0.4
Calcium	910.44 ± 405.16	853.47 ± 382.33	990.99 ± 424.7	*0.01
Magnesium	304.23 ± 122.55	294.63 ± 122.83	317.8 ± 121.56	0.17
Zinc	10.24 ± 5.34	9.69 ± 5.34	11.02 ± 5.29	0.17
Copper	1.67 ± 0.82	1.59 ± 0.82	1.78 ± 0.82	0.09
Selenium	0.04 ± 0.03	0.04 ± 0.03	0.05 ± 0.03	*0.01
Iron	31.02 ± 31.19	29.1 ± 33.92	33.75 ± 26.8	0.28

Values are presented as mean ± SD. A *p*-value of < 0.05 was considered significant. *p < 0.05, **p < 0.01.

## Discussion

In a hospital-based cross-sectional study on 210 infertile men seeking infertility treatment at the referral infertility clinics of Arash and Dr. Shariati hospitals between 2019 and 2021, we found that higher values of dTAC were associated with higher total energy intake, but there were no significant differences in semen parameters or abnormal semen risk across categories of the dTAC score. Similarly, we observed no significant differences in semen parameters across categories of the AHEI score. We found a significant inverse association between DII scores and abnormal semen risk in Q1 after adjusting for total calorie intake, indicating that a more pro-inflammatory diet was associated with lower abnormal semen in the lowest quartile of DII. Additionally, we identified several specific dietary factors inversely associated with semen normality, including total energy, fat, saturated fat, mono-saturated fat, Thiamine, Niacin, and Selenium were associated with abnormal semen.

In this study, before and after adjusting for total calorie intake, there was a significant direct association between DII and the abnormal semen risk, which implies that a pro-inflammatory diet may have a protective effect against abnormal semen. Notably, after adjusting for age, BMI, smoking history, supplement use, and educational status, the association almost remained. While this is inconsistent with prior research which has shown no association between DII scores and asthenozoospermia risk (26). This could be due to variations in factors such as study participants, their habits and behaviors, the outcomes being measured, and the sample sizes used in the studies. In a cross-sectional study on 209 healthy male university students to assess the associations between DII and male reproductive parameters, statistically significant positive associations were observed between the DII and progressive sperm motility and total sperm motility (PR + NP). Nevertheless, there were no significant associations for other semen parameters or male reproductive hormones (34). Thus, a pro-inflammatory dietary status may be associated with increased semen quality. Further research is needed to understand the potential mechanisms and to determine the clinical significance of these findings.

Our results indicate that there is no significant link between dTAC and abnormal semen. This finding is consistent with a previous study by Huang et al. (43), which examined the relationship between dTAC and sperm quality in a case–control study involving 553 patients with asthenozoospermia and 586 men with normal sperm parameters in China. They similarly observed no significant association between dTAC index and the likelihood of developing asthenozoospermia.

We did not find significant differences across categories of AHEI scores regarding semen parameters, nor were there associations between AHEI and the risk of abnormal semen. This is consistent with Cutillas-Tolín et al. (44) who investigated the association of diet quality indices {[AHEI, relative Mediterranean diet score (rMED), and DASH]} with semen quality and hormones involved in reproduction. Although they found meaningful positive associations between the DASH index and sperm concentration, total sperm count, and total motile sperm count, there was no association between AHEI and male reproductive hormones or semen parameters. Meanwhile, according to Efrat et al.’s (25) study, men with the highest quartiles of HEI, AHEI, aMed, and DASH indices had significantly higher adjusted means of sperm concentration, normal sperm morphology, total sperm count, and sperm motility. Specifically, the results showed that the highest quartiles of HEI, AHEI, and DASH were associated with a 10%, 45%, and 24% increase in sperm concentration, respectively. AHEI and DASH were associated with a 21% and 8% increase in normal sperm morphology, respectively, while AHEI was associated with a 29% increase in total sperm count. Moreover, aMed and HEI were associated with a 6% and 11% increase in sperm motility, respectively. The study found that all four dietary indices were positively associated with overall better sperm quality, with AHEI being the most strongly associated. However, we need to be careful not to attribute excessive clinical significance to statistically significant findings. Multicenter studies with larger sample sizes and using more comprehensive dietary assessment methods are necessary to confirm these findings and understand the mechanisms underlying the relationship. Moreover, although semen analysis is crucial in determining men’s fertility capacity, it cannot be considered a reliable predictor of fertility. Therefore, in order to provide more definitive recommendations on the impact of diet on male fertility, further research is required using more accurate indicators of couple fertility, like time to pregnancy for couples attempting natural conception or the likelihood of live birth for couples undergoing ART. With the increasing number of observational studies demonstrating a consistent link between healthy eating habits and improved fertility for both men and women, it becomes necessary to also consider conducting randomized controlled trials (RCTs) that involve dietary interventions (45).

According to our results, total energy, fat, saturated fat, mono-saturated fat, Thiamine (Vitamin B1), Niacin (Vitamin B3), Selenium and Calcium were inversely associated with semen abnormality. There are some potential underlying mechanisms that could explain the observed associations. First, excessive intake of total energy, fat, and saturated fat may lead to obesity, which has been linked to lower sperm quality. Second, inadequate intake of certain nutrients, such as thiamine and niacin, may lead to oxidative stress and inflammation, which can negatively affect sperm production and function. Finally, low intake of calcium may be related to abnormalities in sperm motility and concentration (9, 46, 47). Due to the cultural significance of infertility for men, they pay more attention to their nutrition, and therefore, most likely due to this reason, these values have been higher in our study population.

The Western diet, a dietary pattern rich in many of the nutrients mentioned, has been linked with an elevated risk of male infertility (48). However, following the Mediterranean diet could result in enhanced sperm quality and potentially improved chances of successful pregnancy (38). Lombardo et al. (39) found a statistically significant decrease in inflammatory parameters [leucocytes in seminal fluid and expressed prostate secretion (EPS)] and a notable improvement in progressive sperm motility and sperm morphology in chronic prostatitis patients treated with a dietary supplement containing lycopene, epigallocatechin gallate, ellagic acid, selenium and zincin compared with the untreated group. Also, sperm concentration, motility, and ratio of normal morphology sperms, and total antioxidant capacity increased after a four-month Mediterranean diet and moderate physical activity program in healthy young men (49). According to a prospective cohort conducted by Salas-Huetos (50), although men who followed the Panagiotakos Mediterranean diet and the American Heart Association dietary pattern had a lower fertilization rate, no remarkable differences were observed in semen parameters between individuals in the lower quartile and those in the higher quartile of the eight dietary patterns evaluated. It is important to note that the association between these nutrients and semen quality may vary depending on the source of the nutrients. For example, while high intake of saturated fat from animal sources may be associated with lower sperm quality, intake of monounsaturated fats from plant sources such as olive oil and nuts has been linked to better sperm quality.

Using data from a hospital-based data over 2 years, for the first time, we studied the association between dietary indices (including dTAC, AHEI, and DII scores) and semen parameters among infertile men with normal and abnormal semen seeking for ARTs at two infertility clinics affiliated with Tehran University of Medical Sciences (TUMS). This study would represent a reasonable sample from the Iranian male patients since patients from all over the country visit Arash and Dr. Shariati hospitals, large educational, research, and medical centers which provide comprehensive specialized and subspecialized health care services. A study population consistent in age, ethnicity, and clinical characteristics facilitates the generalizability of the findings to infertile patients in the I.R. Iran. In addition, previous studies have mostly focused on analyzing the effects of single food components or specific food groups on semen quality. Only a limited number of studies have used dietary patterns to examine the relationship between diet and semen quality (51). However, there are some limitations to the study. The cross-sectional nature of the study limits the ability to establish causality and the direction of the observed associations. Furthermore, in a hospital-based study, the accuracy of exposure may be influenced by recall abilities and selection bias. Larger multicenter longitudinal population studies might be warranted to improve the statistical power to confirm the temporal association between dietary factors and semen quality. Potential confounding factors, such as lifestyle factors, environmental exposures, and medical conditions, may influence the observed associations. Our study relied on self-reported dietary intake, which may be subject to recall bias and measurement error. Overall, while the current research on dietary factors and male infertility is novel and promising, further research is needed to address these limitations, to better understand the potential role of dietary interventions in improving male fertility outcomes, and to better understand the underlying mechanisms of the observed associations.

## Conclusion

Overall, certain dietary factors may affect semen quality. The mechanisms underlying the observed associations are likely multifactorial, involving complex interactions between diet, oxidative stress, inflammation, and hormone levels. Further research is required to fully elucidate the mechanisms underlying the associations and to identify specific dietary interventions that may improve male fertility outcomes.

## Data availability statement

The raw data supporting the conclusions of this article will be made available by the authors, without undue reservation.

## Ethics statement

The studies involving humans were approved by Deputy of the Research and Ethics Committee of Tehran University of Medical Sciences. The studies were conducted in accordance with the local legislation and institutional requirements. The participants provided their written informed consent to participate in this study.

## Author contributions

MD: Data curation, Investigation, Methodology, Visualization, Writing – review & editing. NJ: Data curation, Investigation, Methodology, Visualization, Writing – review & editing. ATM: Data curation, Investigation, Methodology, Writing – review & editing. SR: Writing – original draft, Writing – review & editing. FA: Validation, Writing – review & editing. MK: Validation, Writing – review & editing. AT: Resources, Writing – review & editing. MSN: Funding acquisition, Methodology, Project administration, Supervision, Writing – review & editing. AV: Formal Analysis, Project administration, Supervision, Writing – review & editing.
